# Supplementary material for: A Likelihood Approach for Real-Time Calibration of Stochastic Compartmental Epidemic Models
Source: PLoS Comput Biol. 2017 Jan 17;13(1):e1005257. doi: 10.1371/journal.pcbi.1005257 (PMC5240920; doi:10.1371/journal.pcbi.1005257)
Supplement: S1 File — (TAR.GZ) [file pcbi.1005257.s014.tar.gz › HSPH_Online-SI-Revision/Additions/EnKF08/n100-extreme_noisy/n100-extreme_noisy_PF2_EnKF-summary2.pdf]

peak −8	3w spec	3w cum	5w spec	5w cum	end	3w spec LOG	3w cum LOG	5w spec LOG	5w cum LOG	end LOG
mean	1307.2	4942.5	102.4	555.3	65.5	1.1	1.1	1.6	0.7	0.8
median	190.6	1397.5	93.5	209.1	66.5	0.9	1.	1.7	0.7	0.6
quantile .1	84.8	103.6	71.5	80.	31.	0.5	0.3	0.6	0.4	0.2
quantile .9	3778.4	14 144.9	117.4	1125.2	90.5	1.7	2.	2.7	0.4	1.4
22	19	14	21	24						

peak −4	3w spec	3w cum	5w spec	5w cum	end	3w spec LOG	3w cum LOG	5w spec LOG	5w cum LOG	end LOG
mean	91.6	90.	95.7	83.9	83.4	1.8	0.8	2.6	1.	1.1
median	92.5	90.6	96.7	88.4	86.5	1.6	0.8	2.6	1.	1.2
quantile .1	86.6	68.4	91.2	64.4	63.7	1.2	0.3	1.6	0.4	0.6
quantile .9	96.7	105.4	98.8	92.4	92.1	2.7	1.1	3.3	0.4	1.5
4	21	0	14	7						

peak	3w spec	3w cum	5w spec	5w cum	end	3w spec LOG	3w cum LOG	5w spec LOG	5w cum LOG	end LOG
mean	92.	89.3	93.7	88.3	87.5	1.4	0.9	1.7	1.	1.
median	93.1	89.2	95.5	89.1	88.9	1.3	0.9	1.7	0.9	1.
quantile .1	83.8	77.7	83.9	79.3	78.4	0.7	0.6	0.8	0.6	0.6
quantile .9	97.4	98.3	101.8	96.2	95.	2.1	1.3	2.6	0.6	1.4
14	19	12	17	21						

peak +4	3w spec	3w cum	5w spec	5w cum	end	3w spec LOG	3w cum LOG	5w spec LOG	5w cum LOG	end LOG
mean	180.7	209.7	252.4	201.7	205.9	1.1	0.5	1.4	0.5	0.5
median	128.9	168.9	125.5	149.8	139.2	0.8	0.5	1.3	0.5	0.5
quantile .1	71.	83.1	79.8	79.2	83.5	0.4	0.3	0.6	0.3	0.4
quantile .9	280.8	299.5	592.3	279.6	321.5	2.2	0.7	2.4	0.3	0.8
41	47	31	46	46						

peak +8	3w spec	3w cum	5w spec	5w cum	end	3w spec LOG	3w cum LOG	5w spec LOG	5w cum LOG	end LOG
mean	2451.3	1895.9	4480.8	2101.1	2528.4	0.9	0.8	1.1	0.8	0.8
median	515.	670.5	451.3	632.8	552.3	0.7	0.7	1.	0.7	0.7
quantile .1	94.1	156.9	88.1	121.7	109.	0.4	0.4	0.6	0.4	0.4
quantile .9	7819.	5752.9	20 123.2	7356.6	8604.2	1.7	1.3	1.8	0.4	1.5
36	17	41	20	22	{}	{}	{}	{}	{}	{}
